# Diversity and distribution of the tick-borne relapsing fever spirochete *Borrelia turicatae*

**DOI:** 10.1371/journal.pntd.0009868

**Published:** 2021-11-23

**Authors:** Aparna Krishnavajhala, Brittany A. Armstrong, Alexander R. Kneubehl, Sarah M. Gunter, Julie Piccione, Hee J. Kim, Rosa Ramirez, Ivan Castro-Arellano, Walter Roachell, Pete D. Teel, Job E. Lopez

**Affiliations:** 1 Department of Pediatrics and the National School of Tropical Medicine, Baylor College of Medicine, Houston, Texas, United States of America; 2 Texas A&M Veterinary Medical Diagnostic Laboratory, College Station, Texas, United States of America; 3 Department of Entomology, Texas A&M AgriLife Research, College Station, Texas, United States of America; 4 Department of Biology, Texas State University, San Marcos, Texas, United States of America; 5 Public Health Command-Central, Fort Sam Houston, Texas, United States of America; University of Minnesota, UNITED STATES

## Abstract

*Borrelia turicatae* is a causative agent of tick-borne relapsing fever (TBRF) in the subtropics and tropics of the United States and Latin America. Historically, *B*. *turicatae* was thought to be maintained in enzootic cycles in rural areas. However, there is growing evidence that suggests the pathogen has established endemic foci in densely populated regions of Texas. With the growth of homelessness in the state and human activity in city parks, it was important to implement field collection efforts to identify areas where *B*. *turicatae* and its vector circulate. Between 2017 and 2020 we collected *Ornithodoros turicata* ticks in suburban and urban areas including public and private parks and recreational spaces. Ticks were fed on naïve mice and spirochetes were isolated from the blood. Multilocus sequence typing (MLST) was performed on eight newly obtained isolates and included previously reported sequences. The four chromosomal loci targeted for MLST were 16S ribosomal RNA (*rrs*), flagellin B (*flaB*), DNA gyrase B (*gyrB*), and the intergenic spacer (IGS). Given the complexity of *Borrelia* genomes, plasmid diversity was also evaluated. These studies indicate that the IGS locus segregates *B*. *turicatae* into four genomic types and plasmid diversity is extensive between isolates. Furthermore, *B*. *turicatae* and its vector have established endemic foci in parks and recreational areas in densely populated settings of Texas.

## Author summary

In the Americas, the range and genetic diversity of tick-borne relapsing fever spirochetes remains poorly defined. *Borrelia turicatae* is the predominant species in the southwestern and eastern portions of the United States and Latin America, and recent studies suggests the pathogens and their vectors have established endemic foci in highly populated areas of the United States. Given the rate of urban growth and the incorporation of green spaces into large cities, this study supports previous work suggesting the expansion of *B*. *turicatae*.

## Introduction

The genus *Borrelia* is comprise of vector-borne pathogenic spirochetes that cause louse- or tick-borne relapsing fever (TBRF). In the United States, the two predominant species that are associated with human disease are *Borrelia hermsii* and *Borrelia turicatae* [1–4]. *Borrelia hermsii* and its tick vector, *Ornithodoros hermsii*, are distributed in high elevation coniferous forests of the western United States [1]. The range for *B*. *turicatae* and its vector, *Ornithodoros turicata*, is predominantly in the subtropics of the United States and into tropical areas of Latin America [5–7]. Additionally, *B*. *turicatae* presents with nonspecific clinical symptoms and is frequently misdiagnosed as Lyme disease [2, 8, 9], further complicating a clear understanding of its range.

Historically, *B*. *turicatae* and its vector have been identified in rural areas of the United States. *Ornithodoros turicata* ticks have been recovered from California, Nevada, Arizona, Colorado, Utah, New Mexico, Kansas, Oklahoma, Texas, and Florida [5, 7, 10]. However, less is known about the distribution of *B*. *turicatae*. A genetic analysis from an aborted horse fetus indicated that a *B*. *parkeri-B*. *turicatae* type spirochete circulates in California [11], while *B*. *turicatae* was associated with human infections in the Mojave Desert in Nevada [10]. In Texas, *B*. *turicatae* was isolated from field collected ticks, sick domestic canines, and a soldier that was conducing field exercises in the state [12, 13]. There have also been case studies for the distribution of *B*. *turicatae* in rural areas of Texas [9, 14, 15]. In Florida, spirochetes have been isolated from sick canines in the 1990s [12, 16], but little work has followed up on those studies. In these examples, *B*. *turicatae* was primarily identified in low population areas and human infections were associated with field biologists [10], hunters [15], military personnel [13], border patrol [9], and undocumented immigrants [9]. However, over the past five years evidence indicates that *B*. *turicatae* has established endemic foci in urbanized settings.

Recent reports of human exposure to *B*. *turicatae* within the city limits of Austin, Texas indicated the distribution of the pathogen in densely populated areas of Texas. For example, serological surveys of cave workers from Austin showed an occupational hazard to relapsing fever spirochetes in the city [17]. In 2020, a case report of neuroborreliosis caused by *B*. *turicatae* narrowed the suspected exposure site to a park in Austin [18]. Moreover, a 2017 outbreak in Austin was associated with a workshop in the city limits [19]. Follow up studies collecting ticks at a public park nearby the conference center identified another endemic focus of *B*. *turicatae* [2]. Observations during recent field studies also revealed a growing number of camps for homelessness in Austin public parks and greenbelts, and high human activity in and around karst formations in the city. Given the public health relevance of *B*. *turicatae*, there is growing interest to understand the distribution of the pathogen and its vector.

To identify endemic foci for *B*. *turicatae*, centering collection efforts on the tick vector is advantageous. Once *B*. *turicatae* enters a tick population, an endemic focus can quickly become established. *Ornithodoros turicata* serves as a reservoir and host for *B*. *turicatae* since spirochetes are transovarially transmitted to the offspring of infected female ticks [20]. The ticks also live upward of 10 years and endure years of starvation yet transmit *B*. *turicatae* within seconds of attachment [20–22]. Furthermore, *O*. *turicata* ticks can be aggressive feeders once they have detected a bloodmeal source, emerging from under the soil, leaflitter, and the roofs of caves [2, 5, 9, 23]. Consequently, baiting *O*. *turicata* with carbon dioxide improves their collection in attempt to determine whether they are infected.

The objective of this study was to further define the distribution of *B*. *turicatae* in populated areas of central Texas and evaluate the genetic composition of this pathogen. We collected *O*. *turicata* ticks from public and private recreational areas, parks, caves, karst formations, dens, and military installations within the city limits of San Antonio and Austin, the second and fourth largest cities in Texas, respectively. Ticks infectious status was determined by feeding them on naïve mice and culturing spirochetes from the blood. We also cultured spirochetes from the blood of a sick domestic canine that was diagnosed in College Station, Texas. A multilocus sequence typing (MLST) analysis was performed with these spirochete isolates in addition to previously reported human, tick, and canine isolates collected from other locations [12, 24]. Four loci, 16S ribosomal RNA *(rrs)*, flagellin B *(flaB)*, DNA gyrase B *(gyrB*), and the intergenic spacer (IGS) were sequenced totaling 3,050 base pairs per isolate. When available, we incorporated into the phylogenetic analysis previously deposited GenBank sequences. Lastly, we assessed plasmid profiles of 16 isolates. This work continues to identify endemic foci in populated regions of Texas and provides the framework for assessing exposure to *B*. *turicatae* is underserved populations.

## Methods

### Ethics statement

All performed work and animal husbandry was in accordance to the United States Public Health Service policy on Humane Care and Use of Laboratory Animals and the Guide for the Care and Use of Laboratory Animals. Use of mice for field collected tick feedings and transmission feedings was approved by the Baylor College of Medicine (BCM) Institutional Animal Care and Use Committee (protocols AN7086 and AN6563).

### Collection of *O*. *turicata*

Between July 2017 and December 2019, field studies were implemented primarily around San Antonio and Austin, Texas. Field sites were selected based on the accessibility to private land, private and public parks, recreational areas, and green belts (undeveloped land surrounding urban areas). Ticks in the genus *Ornithodoros* were collected from leaf litter at the base of trees, soil inside caves, and subterranean dens where armadillos, woodrats, and ground squirrels were seen. Ticks were CO_2_ baited using dry ice, as previously reported [2], and identified as *O*. *turicata* based on morphological characteristics [7]. Specimens collected from a given location and time point were considered a population. TubeSpin bioreactor tubes (MidSci, St. Louis, MO, USA) were used to house the ticks. In the laboratory, ticks were maintained at 25°C and 85% relative humidity.

### *Borrelia* isolation from ticks

All animal studies were approved by the Baylor College of Medicine Institutional Animal Care and Use Committee (AN7086). Cohorts of ticks, which were a combination of nymphs and adults, were grouped based on collection date and location. To determine if field collected *O*. *turicata* were infectious, four to eight week old female Institute of Cancer Research (ICR) mice (a colony of SWISS origin) were sedated with isoflurane and ~10–20 ticks were placed on the shaved abdomen of the animals and allowed to feed to repletion. A drop of blood was collected from the mice for 10 consecutive days by tail nick and evaluated for the presence of spirochetes by dark field microscopy (Olympus CX33 Trinocular Microscope, Feasterville, PA, USA). Twenty fields were examined using a 20x objective and upon visualizing spirochetes in the blood the mouse was sedated and underwent a terminal cardiac puncture with collection of ~1 mL of whole blood. The blood was centrifuged at 8,000 rpm for 15 minutes and serum was collected. Four ml of modified Barbour-Stonner-Kelly (mBSK)-c medium [25, 26], warmed to 37°C, was inoculated with 50–100 μL of serum. When the spirochetes reached late log phase the cultures were passaged to ensure that they could be successfully cultivated, and were stored in 20% glycerol at -80°C.

### *Borrelia* isolates, genomic DNA extraction, PCR amplification, and MLST

In addition to the isolates obtained in this study, previously reported *B*. *turicatae* isolates were included for comparison purposes (Table 1). This included the 91E135 isolate, which was recovered from murine blood after feeding ticks collected from Crocket County Texas on these laboratory animals [12]. TCB-1 and TCB-2 originated from the blood of two naturally infected, clinically ill dogs from Clay and Lubbock County Texas, respectively [12, 24]. FCB originated from an infected dog from Sumter County Florida [16]. We also included three isolates (BRP1, BRP1a, and BRP2) from prior field studies in Austin, Texas [2], and BTE5EL that originated from a soldier who was conducting training exercises in Texas [13]. All isolates were propagated by taking a portion of frozen glycerol stocks to inoculate 50 mL mBSK-c [25, 26]. Once the spirochetes attained a density of ~1 x 10^7^ spirochetes per ml, genomic DNA was prepared. Genomic DNA of *Borrelia* isolates was extracted using a phenol-chloroform procedure, as previously described [27].

**Table 1:** Designations of *Borrelia turicatae* isolates used in this study and their biological sources, time of collection, and origin.

Isolate Designation & Origin	Host	Isolation Date (Month/Year)	Locality
LCC1, Cave	*O*. *turicata*	12/2017	Bexar Co., San Antonio, TX
MCC1, Cave	*O*. *turicata*	12/2017	Bexar Co., San Antonio, TX
WOC1, Cave	*O*. *turicata*	12/2017	Bexar Co., San Antonio, TX
LOC1, Cave	*O*. *turicata*	6/2017	Travis Co., Austin, TX
LOC3, Cave	*O*. *turicata*	8/2018	Travis Co., Austin, TX
BUC1, Cave	*O*. *turicata*	8/2018	Travis Co., Austin, TX
BRP1*	*O*. *turicata*	7/2017	Travis Co., Austin, TX
BRP1a*	*O*. *turicata*	11/2017	Travis Co., Austin, TX
BRP2*	*O*. *turicata*	11/2017	Travis Co., Austin, TX
LAFB1, Den	*O*. *turicata*	2019	Bexar Co., San Antonio, TX
91E135†	*O*. *turicata*	1991	Crockett Co., Ozona, TX
TCB-1†	Domestic Dog	1999	Clay Co., TX
TCB-2†	Domestic Dog	2001	Lubbock Co., TX
FCB†	Domestic Dog	1992	Sumter Co., FL
CSB, Canine blood	Domestic Dog	3/2020	Brazos Co., College Station, TX
BTE5EL‡	Human Isolate	2015	Martin Co., TX

*Reported by Bissett et al. [2]

†Reported by Schwan et al. [12]

‡ Reported by Christensen et al. [13]

The primers used for PCR amplification of the four *Borrelia* loci are listed in Table 2. PCR conditions for the amplification of *Borrelia rrs*, *flaB*, *gyrB* and IGS loci, amplicon purification, sequencing, and analysis was performed as previously described [13]. For five additional isolates, the sequences for *rrs*, *flaB*, *gyrB*, and IGS were obtained from NCBI.

**Table 2:** Oligonucleotides used for PCR and MLST of relapsing fever spirochetes, Texas, USA*.

Primer	Gene Locus	Sequence (5’-3’)
***rrs***	**16S rRNA**	
UniB†		TACAAGGAGGTGATCCAGC
FD3†		AGAGTTTGATCCTGGCTTAG
16S (+)‡		TACAGGTGCTGCATGGTTGTCG
16S (-)‡		TAGAAGTTCGCCTTCGCCTCTG
Rec4‡		ATGCTAGAAACTGCATGA
P10 REV		ACATAAGGGCCATGATGATT
P6 REV		TTTACAGCGTAGACTACCAG
***flaB***	***flagellin***	
flaB-Exp-For		ATGATCATAAATCATAATACGTCAGCTATAAATG
flaB-Exp-Rev		TCTAAGCAATGATAATACATACTGAGGCAC
flaLL§		ACATATTCAGATGCAGACAGAGGT
flaRL§		GCAATCATAGCCATTGCAGATTGT
***gyrB***	***DNA gyrase***	
gyrB 3’§		GGCTCTTGAAACAATAACAGACATCGC
gyrB 5’+3§		GCTGATGCTGATGTTGATGG
***16S-23S IGS***	***Intergenic spacer***	
IGSF¥		GTATGTTTAGTGAGGGGGGTG
IGSR¥		GGATCATAGCTCAGGTGGTTAG

*For, forward; Rev, reverse.

† Reported by Wang et al [28]

‡ Reported by Porcella et al [29]

§ Reported by Barbour et al [30]

¥ Reported by Bunikis et al [31]

### Phylogenetic analysis

A phylogenetic analysis was performed using sequences from four loci, *rrs*, *flaB*, *gyrB*, and IGS. In addition to the newly acquired *B*. *turicatae* isolates, GenBank sequences were included (S1 Table). These sequences originated from previously published isolates (RML, 95PE-570, 99PE-1807, and PE1-926) [12]. Furthermore, *B*. *turicatae* IGS sequences were included from ticks and clinical canines that originated from Kansas and Texas, respectively [12, 31, 32]. All sequences were aligned using CLUSTAL *W* [33], and phylogenetic trees were generated for each of the four gene sequences using MEGA-X software [34]. A neighbor-joining maximum likelihood method was used to create trees with the isolates. A bootstrap value of 1,000 replicates was used to calculate confidence in tree-drawing parameters [34].

### Field inversion electrophoresis

To assess plasmid composition, field inversion gel electrophoresis was performed. Genomic DNA was used from all the newly acquired and previously published *Borrelia* isolates that were available [12]. Approximately 500 ng of genomic DNA from each isolate was loaded in a 1% Tris Borate EDTA (TBE) agarose gel and separated using the Owl D4 Horizontal Gel Electrophoresis System (Thermo fisher, Waltham, MA, USA) and a PPI-200 programmable power inverter (MJ Research, Inc., Waltham, MA, USA). The gel was run at 100V for 15 minutes and then run at 90V for ~ 40 hours using program 3 of the PPI-200 programmable power inverter.

## Results

### *Ornithodoros turicata* collection sites

**Fig 1:**
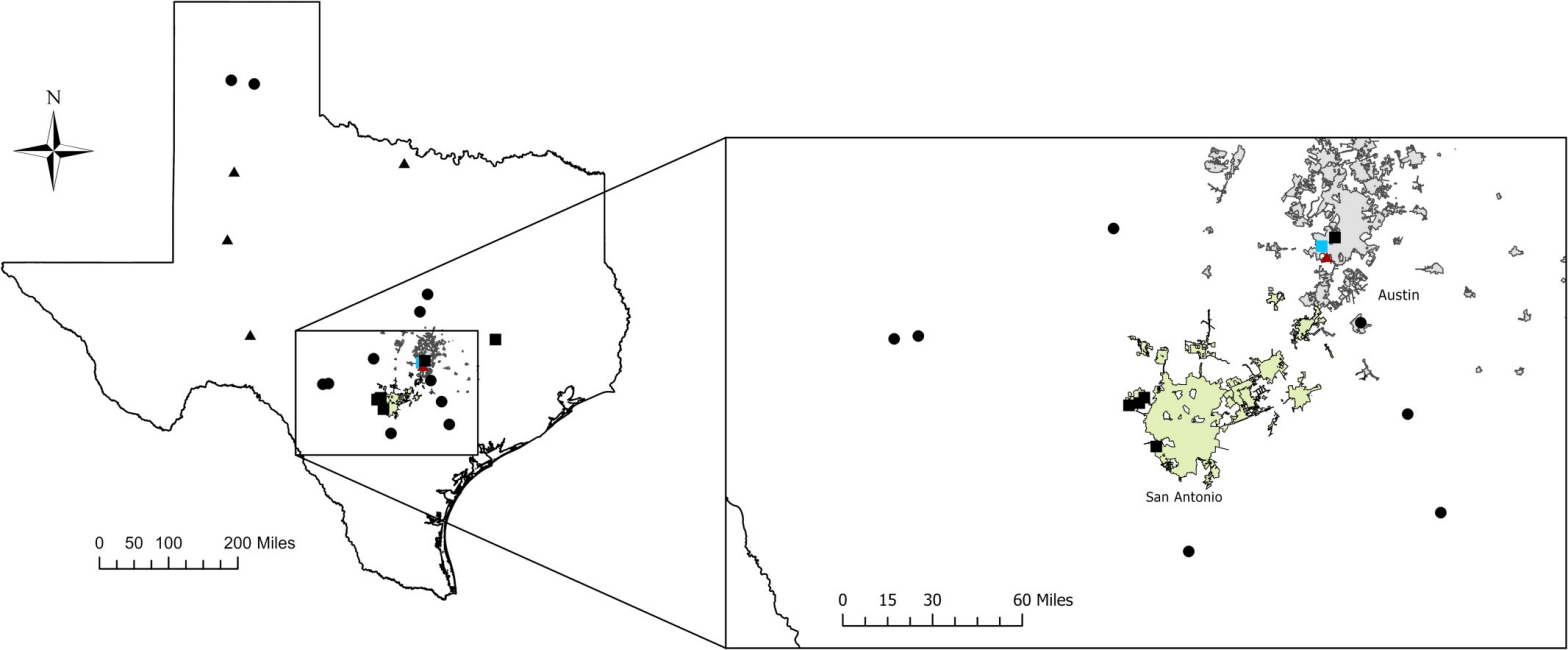
Locality map of *Borrelia turicatae* sequences and isolates used in the study. Sequences originated from newly acquired isolates (squares), previously published isolates (triangles), and sequences obtained from GenBank (circles). The red triangle in the map denotes three collection sites within a 50-meter proximity and blue square indicates two collections from the same location at two different time points. The city limits of San Antonio and Austin, Texas are shown as green and grey, respectively. Scales are shown in the bottom left of the figure and insert. Below are links to the shapefiles used: State outline: https://www.arcgis.com/home/item.html?id=9a43e9a3e2df4a159a57bcef5de96018. Austin city limits: https://regional-open-data-capcog.opendata.arcgis.com. San Antonio city limits: https://geoportal-mpo.opendata.arcgis.com.

Fig 1 shows a map of collection locations of ticks and where DNA sequences originated. *O*. *turicata* were collected in leaf litter, caves, and dens located in public and private locations in Texas. In all but one location, ticks emerged and were collected within ~30 minutes of CO_2_ trap placement. At the collection site in San Antonio, ticks were collected after overnight trapping. The number of collected ticks ranged from 20 to ~100.

### Generation of new *Borrelia* isolates

Tick feedings lasted between 20–60 minutes, and daily evaluation of murine blood by dark field microscopy identified cohorts of infected ticks. Upon inoculating mBSK-c medium with mouse serum, spirochetes were visualized within one week. Spirochetes were passaged into fresh medium once they reached late logarithmic growth, confirming that they could be maintained by *in vitro* cultivation. *Borrelia turicatae* examined in this report included eight new isolates, seven originated from *O*. *turicata* ticks and one from a canine (Table 1). The isolates that originated from ticks include MCC1, WOC1, LCC1, LOC1, LOC3, BUC1, and LAFB1, (Table 1). WOC1 and MCC1 originated from ticks that were collected in caves that were ~150 m apart. Ticks infected with the LCC1 isolate were ~ 835 m from the ticks that produced WOC1 and MCC1. LOC1 and LOC3 were obtained from single cave from Austin, Texas, while BUC1 was obtained from ticks from a separate cave 6.4 km away. LAFB1 was collected from a *Neotoma* species nest. The canine isolate, CSB, originated from a dog that presented to their veterinarian for lethargy, inappetence, hesitance to walk, and leg tremors. The clinical work-up revealed fever, mild anemia, lymphopenia, marked thrombocytopenia, and visible spirochetemia on standard blood film.

### Genetic analysis of partial 16S rRNA *(rrs)*, *flaB*, *gyrB* and IGS loci

For the phylogenetic analysis, 16 isolates were evaluated with additional *B*. *turicatae* sequences obtained from GenBank. For example, for the *rrs*, *flaB*, and *gyrB* loci, three additional sequences were included for each locus. These GenBank sequences originated from the previously reported PE1-926, 99PE-1807, and 95PE-570 isolates of *B*. *turicatae* [12]. For the IGS locus, we included GenBank sequences from PE1-926, 99PE-1807, 95PE-570, an isolate that originated in Kansas (RML), and eight *B*. *turicatae* sequences from TBRF positive canines [12, 31, 32]. Accession numbers for all sequences used in the study are shown in the S1 Table.

The phylograms produced from *rrs*, *flaB*, and *gyrB* generated little variation at these loci. A comparative analysis of the 1,203 bp *rrs* gene sequences produced two alleles (accession numbers in S1 Table). The FCB isolate had a single nucleotide substitution at position 1,029 while the sequences from the remaining isolates were identical.

Sequence analysis of the 813 bp *flaB* locus produced little variation between amplicons (accession numbers in S1 Table). Comparison of the sequences grouped 16 together. FCB, WOC1, MCC1 differed from rest of the sequences by a single nucleotide at positions 306, 710, and 885, respectively.

Sequence comparisons indicated that *gyrB* is highly conserved among all *B*. *turicatae* sequences evaluated. Comparison of the 369 bp amplicon from the *gyrB* locus produced two alleles in the analyzed sequences (accession numbers in S1 Table). LCC1, MCC1, and WOC1 grouped together and differed at position 259 and the remaining sequences formed a single group.

**Fig 2:**
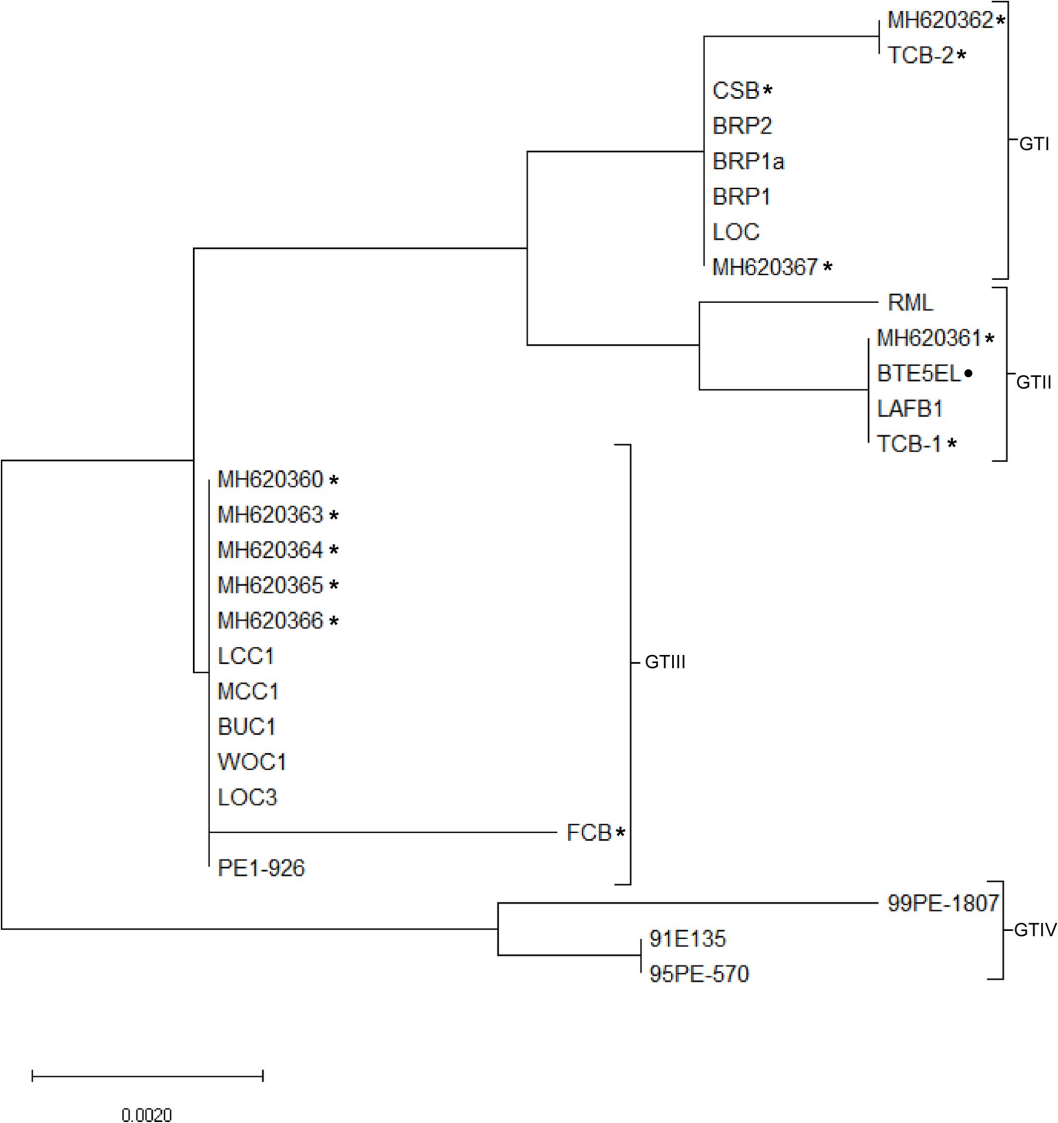
Phylogram of the intergenic spacer sequences from *Borrelia turicatae*. Sequences were obtained from infected canines (*), a human (•), and all others *B*. *turicatae* IGS sequences originated from infected ticks. The phylogenetic tree was generated with 1,000 bootstrap replicates. The scale bar for branch lengths represents the number of nucleotide substitutions per site (0.0020).

To genetically type the isolates, the 665-bp IGS locus was evaluated. Alignments of the 28 sequences indicated nucleotide differences at 15 positions: 56, 67, 97, 99, 104, 136, 155, 203, 323, 328, 335, 392, 396, 442 and 455. Furthermore, the IGS loci organized the 28 *B*. *turicatae* IGS sequences into four genotypes (GTI-IV) (Fig 2). Within a genotype, multiple alleles were identified. For example, GTI contained eight sequences and produced two alleles, with BRP2, CSB, BRP1a, BRP1 and LOC1 and accession number MH620367 being identical and differing from TCB-2 and accession number MH620362 by 1 base at position 396 (Fig 2 and S1 Table). GTII produced two alleles with one consisting of *B*. *turicatae* originating from Kansas. The other GTII allele consisted of accession number MH620361, BTE5EL, LAFB1, and TCB-1 (Fig 2 and S1 Table). These two alleles differed at positions 97 and 455. GTIII clustered into two alleles with LCC1, MCC1, BUC1, WOC1, LOC1, PE1-926, and accession numbers MH620360 and MH620363-MH620366 being identical and differing from FCB by two nucleotides at positions 56 and 323 (Fig 2 and S1 Table). GTIV clustered into two alleles with 91E135, 95PE-570 being identical and differing from 99PE-1807 at nucleotide positions 203, 392 and 455 (Fig 2). IGS sequence analysis identified unique genotypes in the compared sequences, and these findings indicated that the locus was more polymorphic compared to *rrs*, *flaB*, and *gyrB*.

### Plasmid assessment of *B*. *turicatae*

**Fig 3:**
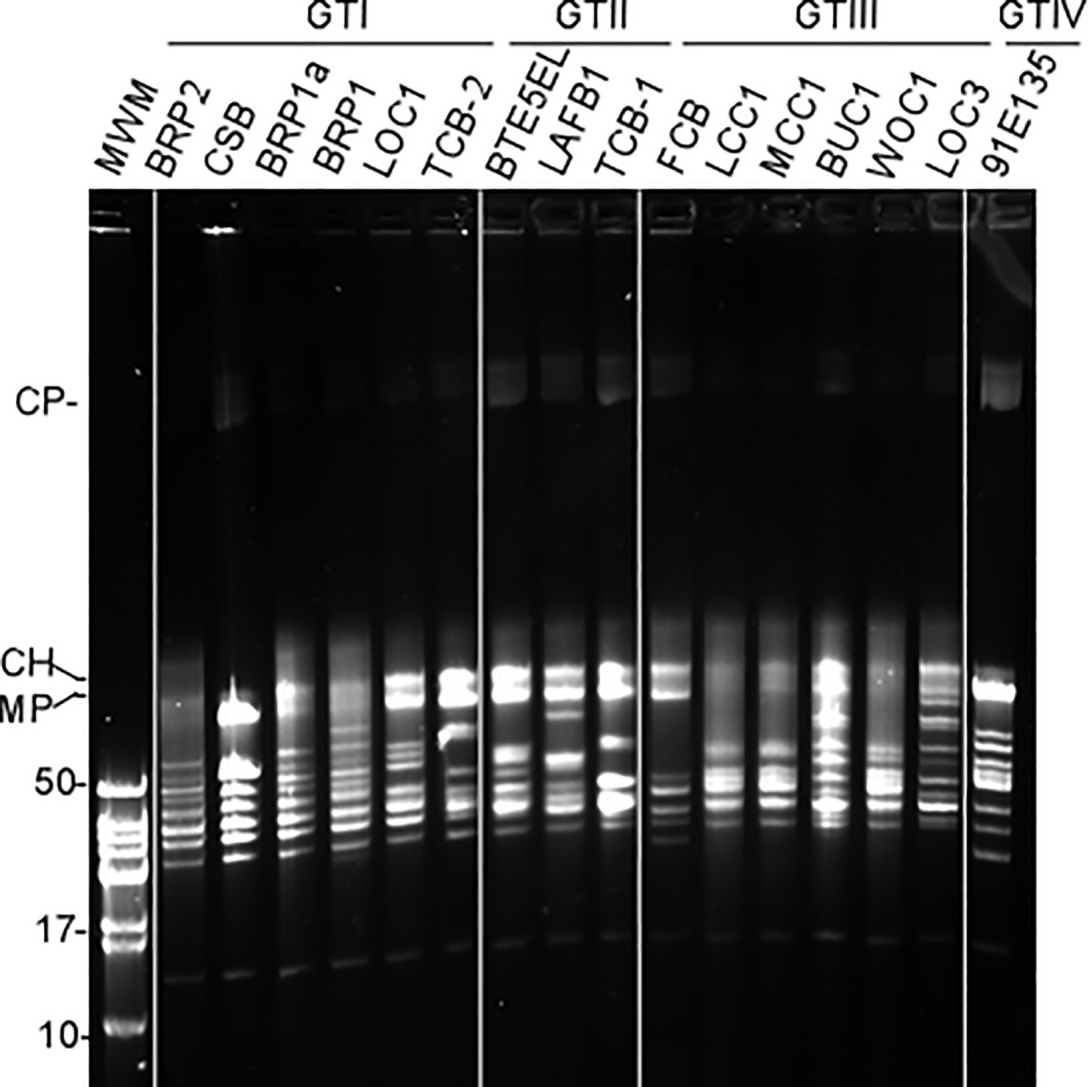
Plasmid profiles of 16 *Borrelia turicatae* isolates from Texas and Florida. Shown are previously reported and new isolates of *Borrelia turicatae*. The isolate designations are shown on top and are organized by genotype. Molecular size standards (MSS) in kilobases are shown on the left. CP: circular plasmid; CH: chromosome; MP: megaplasmid.

*Borrelia* genomes are complex with multiple linear and circular plasmids that vary among species and within strains [12]. Performing field inversion gel electrophoresis identified the linear chromosome, megaplasmid, and a circular plasmid from all the isolates (Fig 3). They also contained a small linear plasmid of ~13 kb and 8–12 linear plasmids ranging from ~20–80 kb. Moreover, plasmid diversity between the four genotypes of *B*. *turicatae* was observed.

Plasmid maps of GTI *B*. *turicatae* isolates had approximately eight to 11 linear plasmids each ranging from ~13–80 kb (Fig 3). Plasmid profiles of BRP1, BRP1a and BRP2 looked similar except that BRP1 contained additional ~20 kb and ~80 kb plasmids, as previously reported [2]. We did not detect a ~50 kb plasmid in CSB, while LOC1 lacked a ~45 kb plasmid compared to BRP isolates. Plasmid organization of TCB-2 differed from other GTI isolates in that it contains two distinct plasmids at ~35 kb and ~75 kb but lack plasmids ~46 kb, ~50 kb and 80 kb.

We detected seven to 10 linear plasmids ranging from ~13–85 kb in *B*. *turicatae* GTII isolates. The BTE5EL isolate had ~10 plasmids while LAFB1 and TCB-1 each had 8. LAFB1 contains two distinct ~36 kb and ~85 kb plasmids and lack a ~35 kb, ~38 kb, ~46 kb and ~48 kb plasmid compared to the BTE5EL isolate. Similarly, the TCB-1 isolate contained a distinct ~29 kb plasmid and lacked a ~44 kb and ~45 kb plasmid.

Plasmid organization of *B*. *turicatae* GTIII indicated seven to 11 plasmids each ranging from ~13–100 kb. Plasmid profiles of PE1-926 were not included because we did not have bacterial stocks to generate gDNA preparations. Plasmid maps of BUC1 and LOC3 were identical except that they contained a ~20 kb and 90 kb plasmid, respectively. Plasmid maps of LCC1, MCC1 and WOC1 isolates (obtained from same local area) indicated fewer plasmids with variability in plasmid profiles compared to BUC1 and LOC3. They contained distinct ~38 and ~48 kb plasmids and lacked a ~24 kb, ~55 kb, and a ~60 kb plasmid. FCB isolate contained distinct ~20 kb, ~29 kb, ~38 kb, and a ~95 kb plasmids, while the ~13 kb, ~24 kb, and ~33 kb plasmids were similar in size to those detected in BUC1 and LOC3.

GTIV consisted of 91E135, 95PE-570 and 99PE-1807 isolates and a plasmid profile for only the 91E135 isolate was evaluated because we did not have the bacterial stocks for the other two isolates to generate gDNA. From the analysis of 91E135, 11 plasmids were detected. Collectively, with all the isolates evaluated we observed considerable variability in number and size of linear plasmids.

## Discussion

The objective of this study was to identify endemic foci of *B*. *turicatae* primarily in densely populated areas of central Texas and evaluate the genetic composition of the pathogens. We expanded the number of available isolates, which are crucial to address the ecology and pathogenesis of *B*. *turicatae*. Most of our isolates originated from ticks that were collected in public parks and green spaces within metropolitan areas of Texas. MLST indicated that three chromosomal loci (*rrs*, *flaB* and *gyrB*) were highly conserved, while typing of a fourth locus (IGS) demonstrated sequence polymorphism and separated *B*. *turicatae* into four genotypes.

Studies in other species of TBRF spirochetes indicate the spirochetes segregate into genomic groups. This was first reported for *B*. *hermsii* when MLST of over 30 isolates was performed targeting the chromosomal loci *rrs*, *flaB*, *gyrB*, and *glpQ* [29]. Regardless of whether the sequences were concatenated or individually analyzed, *B*. *hermsii* isolates separated into two genomic groups (GGI and GGII) [29]. A genetic analysis of the IGS locus grouped over 40 *B*. *hermsii* isolates into GGI or GGII [3]. Similarly, MLST of the Old World TBRF species *Borrelia hispanica* separated six isolates into two genomic groupings [35]. In that study, seven loci (*rrs*, *flaB*, IGS, *p66*, *groEL*, *glpQ*, and *recC*) were sequenced, and phylogenetic analyses from all loci except the *rrs* generated GGI and GGII clusters [35]. A primary difference we observed with *B*. *turicatae* was the high degree of homogeneity between *rrs*, *flaB*, and *gryB*. Given this observation, our approach was adjusted to evaluate IGS. Our findings supported prior work by Bunikis and co-workers indicating that the 16S-23S IGS region is sensitive and sufficient for TBRF spirochete genotyping [31]. While *rrs*, *flaB*, and *gyrB* have been used in other species of TBRF spirochete for genetic typing, IGS was the only locus that provided the resolution to separate and type *B*. *turicatae* isolates.

The intraspecies homogeneity observed with *B*. *turicatae* suggested that dispersal of *B*. *turicatae* in nature may be complex compared to the rodent-tick enzootic cycle observed in most other species of TBRF [4, 36, 37]. For example, BTE5EL, LAFB1, TCB-1, and the GenBank sequence MH620361, which grouped together as GTII, were obtained from samples that originated over 300 miles apart. There is supportive evidence that migratory vertebrates are involved in the dispersal of *B*. *turicatae* in nature [5]. For example, most isolates that originated from nonhuman vertebrates have come from domestic canines [12, 38]. This has caused us to suspect wild canids in the distribution of *B*. *turicatae*. In support of this, *O*. *turicata* ticks have been collected in dens and caves frequented by coyotes [5]. Furthermore, a serosurveillance study indicated grey foxes and coyotes are likely involved in the spirochete’s ecology [14]. Sera was acquired from terminally sampled animals and antibody reactivity was determined to *B*. *turicatae* protein lysates and the *Borrelia* immunogenic protein A (BipA) [14], a diagnostic antigen used for TRBF spirochetes [39, 40]. The work determined that ~11% of sampled coyotes had circulating antibodies to recombinant BipA and *B*. *turicatae* while serological responses were also detected from grey fox. Coyotes establish social structures and are classified as resident or transient. Resident coyotes (females, dominant males, juveniles and pups) possess a home range of 8 km^2^ to 29 km^2^ [41, 42], while subordinate coyotes exhibit a wider home range of 40 km^2^ to 395 km^2^ [41, 42]. The broad home ranges of coyotes may provide a mechanism for the dispersal of *B*. *turicatae* and its vector

Additional hosts that *O*. *turicata* feeds upon and that may have a role in the ecology and dispersal of *B*. *turicatae* include wild pigs, owls, and bats[5, 43, 44], and a commonality shared by these vertebrates is their utilization of caves and karst formations for shelter [5, 45–47]. An analysis of the Texas Speleological Survey indicates that Travis, Hays, Williamson, and Bexar Counties, which establish the corridor between Austin and San Antonio, possess over 5,100 karst formations [48]. Given that caves and karst formations are known to harbor an immense amount of biodiversity [49], they likely serve as foci for the introduction, maintenance, and dispersal of *B*. *turicatae*.

The identification of genotypes and the plasmid diversity observed between isolates may provide insight into how *B*. *turicatae* is maintained in nature. Plasmid profiles showed that each isolate contained ~seven to 11 plasmids ranging from ~13–150 kb. Furthermore, evidence of multiple genotypes circulating within a region was apparent with LOC1 (GT1) and LOC3 (GTIII). The ticks that produced the LOC1 and LOC3 isolates originated from the same location but were collected at two different time points. LOC1 and LOC3 contained about eight and ~12 plasmids, respectively. Given that *O*. *turicata* are indiscriminate feeders, future studies should evaluate the competence of different vertebrate host species to *B*. *turicatae* genotypes.

A limitation of this study was that we did not determine the prevalence of ticks infected with *B*. *turicatae* and our method of isolation from mice could skew population structures. Until recently, there were approximately eight isolates of *B*. *turicatae* to work with in the laboratory [12]. We reasoned that initial field studies should focus on addressing this short coming, and this current study and recent work has doubled the number of laboratory isolates of *B*. *turicatae* [2]. Moreover, utilization of PCR to detect *B*. *turicatae* DNA in individual ticks is inconsistent. Another limitation was that all but the CSB isolate originated from laboratory mice that were fed upon by field collected ticks. While this approach was the most practical way to identify endemic foci for *B*. *turicatae*, obtaining isolates directly from human and wildlife sources could provide a more refined understanding of *B*. *turicatae* populations that have adapted to specific hosts. This would also identify vertebrate hosts that are currently unknown. For example, one collection site was a *Neotoma* nest, but it remains unknown if these animals are competent hosts for *B*. *turicatae*.

Our results serve as the basis for developing larger-scale investigations into TBRF spirochete vector-host competence, ecology, epidemiology, and the disease’s impact on resource limited populations. While the number of isolates obtained in this work significantly expanded, most sequences originated from Texas. Given the vector’s widespread distribution across the southern United States and into Latin America, more isolates are needed to understand the maintenance and prevalence TBRF spirochetes. Concentrating collection efforts in parks and recreational areas will identify endemic foci where the disease may be misdiagnosed and overlooked. Furthermore, given the widespread distribution of *O*. *turicata* into Latin America, our findings support further investigations on the circulation of TBRF spirochetes impacting the impoverished [8].

## Supporting information

S1 Table NCBI accession numbers of the four genes sequenced from all *B*. *turicatae* isolates and sequences used in this study. (DOCX)
