# Supplementary material for: Diversity and distribution of the tick-borne relapsing fever spirochete Borrelia turicatae
Source: PLoS Negl Trop Dis. 2021 Nov 23;15(11):e0009868. doi: 10.1371/journal.pntd.0009868 (PMC8651100; doi:10.1371/journal.pntd.0009868)
Supplement: S1 Table — (DOCX) [file pntd.0009868.s001.docx]

S1 Table. NCBI accession numbers of the four genes sequenced from all

*B. turicatae* isolates and sequences used in this study.

|  | **Locus** | | | |
| --- | --- | --- | --- | --- |
| **Origin of material** | ***rrs*** | ***gyrB*** | ***flaB*** | **IGS** |
|  |  |  |  |  |
| LCC1 | MT633567 | MT880226 | MT890146 | MT682898 |
| MCC1 | MT633568 | MT880227 | MT890147 | MT682899 |
| WOC1 | MT633569 | MT880228 | MT890148 | MT682900 |
| LOC1 | MT633570 | MT880229 | MT890149 | MT682901 |
| LOC3 | MT633571 | MT880230 | MT890150 | MT682902 |
| BUC1 | MT633573 | MT880231 | MT890151 | MT682903 |
| BTE5EL | MT633572 | MT880232 | MT890152 | MT682907 |
| LAFB1 | MT633574 | MT880233 | MT890153 | MT682908 |
| CSB | MT633575 | MT880234 | MT890154 | MT682909 |
| BRP1 | MH503950 | MH503951 | MH503949 | MT682904 |
| BRP1a | MH507600 | MH507601 | MH507599 | MT682905 |
| BRP2 | MH507603 | MH507604 | MH507602 | MT682906 |
| TCB1 | AY934609.1 | AY934616.1 | AY934628.1 | DQ855554.1 |
| TCB2 | AY934610.1 | AY934617.1 | AY934629.1 | [DQ855558.1](https://www.ncbi.nlm.nih.gov/nucleotide/DQ855558.1?report=genbank&log$=nuclalign&blast_rank=17&RID=SKXNFEPK014) |
| FCB | L37837.1 | AY934618.1 | AY934630.1 | [DQ855552.1](https://www.ncbi.nlm.nih.gov/nucleotide/DQ855552.1?report=genbank&log$=nuclalign&blast_rank=16&RID=SM01AWRB016) |
| 91E135 | AY604974.1 | AY934612.1 | AY604979.1 | DQ855557.1 |
| 95PE-570 | AY934606.1 | AY934613.1 | AY9345625 | DQ855553 |
| PE1-926 | AY934608.1 | AY934615.1 | AY934627.1 | DQ855555 |
| 99PE-1807 | AY934607.1 | AY934614.1 | AY934626.1 | DQ855556 |
| RML | AY934605.1 | AY934611.1 | NA | AY526495 |
| Canine 1 | NA | NA | NA | MH620360 |
| Canine 2 | NA | NA | NA | MH620361 |
| Canine 3 | NA | NA | NA | MH620362 |
| Canine 4 | NA | NA | NA | MH620363 |
| Canine 5 | NA | NA | NA | MH620364 |
| Canine 6 | NA | NA | NA | MH620365 |
| Canine 7 | NA | NA | NA | MH620366 |
| Canine 8 | NA | NA | NA | MH620367 |

NA Not available
